# Frequency of PSA-mRNA-bearing cells in the peripheral blood of patients after prostate biopsy

**DOI:** 10.1054/bjoc.2001.1924

**Published:** 2001-08

**Authors:** N Hara, T Kasahara, T Kawasaki, V Bilim, Y Tomita, K Obara, K Takahashi

**Affiliations:** Division of Molecular Oncology Department of Signal Transduction Research; Division of Urology, Department of Regenerative and Transplant Medicine, Niigata University Graduate School of Medical and Dental Science, Asahimachi 1, Niigata, 951-8510, Japan

**Keywords:** RT-PCR, PSA-mRNA bearing cells, prostate cancer

## Abstract

Transrectal ultrasound (TRUS) guided prostate biopsy is standard diagnostic procedure for prostate cancer (PCa). However, possibility of dissemination of cancer cells by biopsy is not negligible. To investigate this possibility, we examined prostate specific antigen (PSA)-bearing cells in peripheral blood of the 108 patients before and after prostate biopsy. Peripheral blood samples were obtained from 108 patients with elevated serum PSA (sPSA) levels, who had undergone sextant prostate biopsy using TRUS. The presence of PSA-mRNA bearing cells was examined using the nested RT-PCR method enabling detection of one LNCaP cell diluted in 1 ml of whole blood. Among 108 patients, 62 and 46 were diagnosed with benign prostatic hyperplasia (BPH) and PCa, respectively. PSA-mRNA was detected in 3 PCa cases but in no BPH patients before and after biopsy, and in 16 BPH (25.8%) and in 21 PCa (45.7%) patients only after biopsy (*P*< 0.01). The patients with positive mRNA before biopsy had higher sPSA (*P*< 0.001), and those after biopsy had higher sPSA and PSA density (PSAD) levels (*P*< 0.05). Positive PSA-mRNA cases had more cancer involved biopsy cores than the negative PSA-mRNA cases (*P*< 0.001). Although further investigations are needed, the present findings suggest that prostate biopsy might scatter prostate cells in the blood stream especially in cases with high sPSA and, thus, might contribute to tumour spreading in the cases of prostate cancer. © 2001 Cancer Research Campaign http://www.bjcancer.com


*British Journal of Cancer* (2001) **85**(4), 557–562
© 2001 Cancer Research Campaign
doi: 10.1054/ bjoc.2001.1924, available online at http://www.idealibrary.com on IDEAL

http://www.bjcancer.com

### Frequency of PSA-mRNA-bearing cells in the peripheral blood of patients after prostate biopsy

**N Hara[1], T Kasahara[1], T Kawasaki[1], V Bilim[1], Y Tomita[1], K Obara[2] and K Takahashi[2]**

[1]Division of Molecular Oncology, Department of Signal Transduction Research and [2]Division of Urology, Department of Regenerative and Transplant Medicine, Niigata University Graduate School of Medical and Dental Science, Asahimachi 1, Niigata 951-8510, Japan

**Summary** Transrectal ultrasound (TRUS) guided prostate biopsy is standard diagnostic procedure for prostate cancer (PCa). However, possibility of dissemination of cancer cells by biopsy is not negligible. To investigate this possibility, we examined prostate specific antigen (PSA)-bearing cells in peripheral blood of the 108 patients before and after prostate biopsy. Peripheral blood samples were obtained from 108 patients with elevated serum PSA (sPSA) levels, who had undergone sextant prostate biopsy using TRUS. The presence of PSA-mRNA bearing cells was examined using the nested RT-PCR method enabling detection of one LNCaP cell diluted in 1 ml of whole blood. Among 108 patients, 62 and 46 were diagnosed with benign prostatic hyperplasia (BPH) and PCa, respectively. PSA-mRNA was detected in 3 PCa cases but in no BPH patients before and after biopsy, and in 16 BPH (25.8%) and in 21 PCa (45.7%) patients only after biopsy ($P < 0.01$). The patients with positive mRNA before biopsy had higher sPSA ($P < 0.001$), and those after biopsy had higher sPSA and PSA density (PSAD) levels ($P < 0.05$). Positive PSA-mRNA cases had more cancer involved biopsy cores than the negative PSA-mRNA cases ($P < 0.001$). Although further investigations are needed, the present findings suggest that prostate biopsy might scatter prostate cells in the blood stream especially in cases with high sPSA and, thus, might contribute to tumour spreading in the cases of prostate cancer. © 2001 Cancer Research Campaign http://www.bjcancer.com

**Keywords**: RT-PCR; PSA-mRNA bearing cells; prostate cancer

Prostate cancer (PCa) is the second leading cause of cancer-related death in men in the United States (Greenlee et al, 2001) and also is a common cancer increasing in Japan (Yamamoto, 1999). Current staging modalities in predicting locally advanced and metastatic PCa include serum prostate-specific antigen (sPSA) level, transrectal ultrasound (TRUS), computed tomography (CT) scan (Catalona et al, 1994). PSA, which belongs to the tissue kallikrein family, is primary produced by the prostatic epithelium and the secretion is regulated by the androgen level (Webber et al, 1996). PSA secretion is tissue specific, and elevated sPSA is an effective marker for identifying men who have PCa (Crawford et al, 1997). However, it is also often elevated in men with benign prostatic hyperplasia (BPH), prostatitis and other non-malignant disorders (Crawford and DeAntoni, 1993). Therefore, to obtain clinical diagnosis, prostate biopsy is performed. However, in other urological tumours like renal cell cancer (RCC), biopsy is not a common modality for diagnosis, since it might cause cancer cells dissemination into blood flow or surrounding tissues and contribute to disease progression (Smith, 1984; Kiser et al, 1986). This point is not negligible also when regarding prostate cancer, and the implication in biopsy and possible tumour dissemination needs to be investigated. Despite the clinicopathological stage of the disease in the patients with PCa, the frequency of detection of distant metastasis by routine clinical analysis is not concordant with the frequency of disease recurrence after definitive local therapy (Levine et al, 1986).

*Received 3 January 2001*
*Revised 9 April 2001*
*Accepted 1 May 2001*

*Correspondence to:* Y Tomita

Reverse transcription polymerase chain reaction (RT-PCR) is a sensitive and useful method for detecting the presence of a specific cell type such as those bearing tumour-specific mRNA. Recently, several studies reported the presence of PSA-mRNA bearing cells, most likely PCa cells, in the peripheral blood using RT-PCR. Their detection rates in PCa patients were reported as 0 ~ 88% (Gomella et al, 1997; Corey and Corey 1998; Fadlon and Hamdy, 1998; Berteau et al, 1999; de la Taille et al, 1999). Although it has been reported that prostate massage, digital rectal examination and ejaculation raise sPSA levels to some extent (Yuan et al, 1992; Zisman et al, 1997), the presence of PSA-mRNA-bearing cells in the peripheral blood following these events has been seldom reported. Furthermore, the higher frequency of PSA-mRNA-bearing cells in the peripheral blood was correlated with the extent of the disease (de la Taille et al, 1999; Mejean et al, 2000; Su et al, 2000). Thus, the presence of PSA-mRNA-bearing cells in peripheral blood after biopsy might contribute to tumour progression leading to poorer prognosis. In the present study, we further examined the relationship between clinicopathological findings and those detected using nested RT-PCR in prostate cancer patient's peripheral blood.

### MATERIALS AND METHODS

### Patients and their clinicopathological characteristics

A total of 108 patients with serum PSA levels elevated more than 4.0 ng $ml^{-1}$ were selected for this study. Patient characteristics are presented in Table 1. Informed consent to perform the study was obtained from all patients. Systemic sextant prostate biopsy was performed using transrectal ultrasound between October, 1998 and April, 2000, and if a hypoechoic lesion or hypervascular lesion was seen, an additional directed biopsy was performed when

**Table 1** Patient characteristics

| | Number of patients | Age (mean) y. o. | sPSA (mean) ng $ml^{-1}$ | P. volume (mean) $cm^3$ | PSAD (mean) ng $ml^{-1}$ $cm^{-3}$ |
|---|---|---|---|---|---|
| BPH | 62 | 52–90 (70) | 4.0–32.5 (8.9)* | 14.2–120 (38.6) | 0.08–1.22 (0.23)* |
| PCa | 46 | 42–90 (72) | 4.0–1121 (72.1) | 15.3–72.0 (42.2) | 0.08–15.8 (1.71) |
| Total | 108 | 42–90 (70) | 4.0–121 (35.8) | 14.1–120 (39.6) | 0.08–15.8 (0.86) |

*$P < 0.05$.

needed. Disease-specific survival and disease progression-free survival were investigated in March 2001, and the mean observation periods were 17.8 and 17.1 months, respectively.

### Blood samples and total RNA extraction

Peripheral blood samples were obtained before and within 10 minutes after prostate biopsy. Total RNA was extracted from 1 ml of whole blood anticoagulated with EDTA using an SV total RNA isolation system (Promega, Madison, Wisconsin).

### RT-PCR

Nested PCR using outer and inner primer sets were applied. The sequences used for external PSA 20-mer primers were obtained from GenBank and were selected for lowest homology with human kallikrein nucleotide sequences examined by BLAST search. Outer primers were previously reported to identify circulating prostate tumor cells in blood (Ferrari et al, 1997). They were anchored in exon 3 at nucleotides 648–667 (5′-GATGACTCCAGCCAC-GACCT-3′) and in exon 5 at nucleotides 1357–1338 (5′-CACAGA-CACCCCATCCTATC-3′). The nested PSA 20-mer primers were designed to be anchored in exon 4 at nucleotides 860–879 (5′-GCAAGTTCACCCTGAGAAGG-3′) and in exon 5 at nucleotides 1315–1296 (5′-GATATGTCTCCAGGCATGGC-3′).

The first strand DNA was synthesized using a single strand cDNA synthesis kit (Boehringer Mannheim, Inc, Mannheim, Germany) according to the manufacturer's instructions. Resulting cDNA was amplified by nested PCR in a thermal cycler 9600 (Perkin-Elmer, Norwalk, Connecticut). We amended the RT-PCR methods reported previously (Saito et al, 1996; Ferrari et al, 1997).

To control for RNA integrity and to determine the significance of a negative PCR assay, we performed an RT-PCR assay involving the product of the housekeeping gene beta-actin. A set of 20-mer primers anchored at nucleotides 416–435 (5′-GATAT-GTCTCCAGGCATGGC-3′) and nucleotides 859–840 (5′-GATATGTCTCCAGGCATGGC-3′) of the beta-actin cDNA was synthesized to amplify a fragment of 520 bp.

3 negative control reactions were included in the PSA PCR experiment – a reaction with no added cDNA and a reaction containing human female peripheral blood mononuclear cells cDNA and that of healthy young males. A reaction containing cDNA synthesized from RNA derived from a mixture of one LNCaP cell and 1 ml of whole peripheral blood served as a positive control in each experiment. Dilutions of LNCaP were prepared as follows. LNCaP cells were counted in a chamber, and 1000 and 100 cells were directly diluted in 1 ml of whole blood anticoagulated with EDTA. 100 cells were further subjected to serial dilutions to furnish 10 cells and 1 cell in 1 ml. To confirm the reproducibility of the procedure, it was repeated 5 times which demonstrated same results, one of which is represented in Figure 1.

### Sequencing

The PCR products were electrophoresed in agarose gel electrophoresis and stained with ethidium bromide to confirm the products were derived from PSA-mRNA, the nucleotide sequences of some PCR products were sequenced. Briefly, purified products from agarose gel using Suprec 01 DNA recovery tubes (Takara, Japan), were directly determined on an ABI Prism 310 Genetic Analyzer (Perkin Elmer Japan Applied Biosystems). Labeling was performed with an inner PCR primer and a BigDye Terminator RR Mix (Perkin Elmer Japan Applied Biosystems). Each sequence was verified in both the sense and antisense directions (data not shown).

### Statistical analysis

Statistical comparisons were made using the Welch corrected *t*-test and $\chi^2$ tests in StatView 5.0 software for a Macintosh computer. Survival curves were generated using the method of Kaplan and Meier, and differences between curves were evaluated using the log-rank test.

### RESULTS

### PSA RT-PCR analysis of peripheral blood samples

RNA was extracted from the peripheral blood of 119 patients before and after prostate biopsy. As a first step, beta-actin PCR

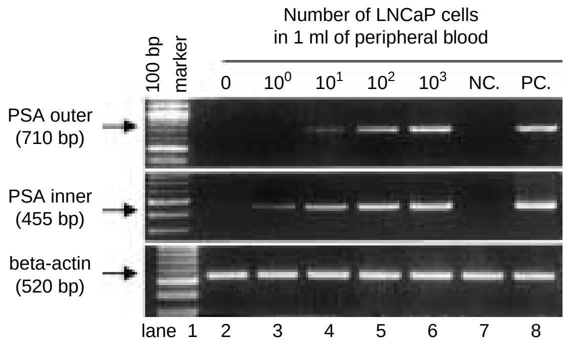

**Figure 1** Detection of PSA-mRNA by single (upper panel – 710 bp) and nested (middle panel – 455 bp) RT-PCR. Beta-actin as a control mRNA (lower panel – 520 bp). Lane 1, molecular weight marker. Zero (lane 2), One (lane 3), 10 (lane 4), 100 (lane 5) and 1000 (lane 6) LNCaP human PCa cells were mixed with 1 ml of human female peripheral blood and amplified by RT-PCR. Zero (lane 2) was one of the negative controls and NC (lane 7) was also a negative control with a reaction containing peripheral blood mononuclear cells cDNA of a healthy young male. Another negative control without cDNA was prepared (data not shown). PC (lane 8) was a positive control

 © 2001 Cancer Research Campaign assay was performed to evaluate RNA integrity. 11 cases were excluded because of degradation of RNA. RNA samples from 108 patients were examined using RT-PCR. PSA-mRNA was detected in 3 PCa but in no BPH cases before biopsy. In 37 cases (16 of 62 BPH cases (25.8%) and 21 of 46 PCa cases (45.7%), PSA-mRNA was detected only after biopsy. There was no case, in which PSA-mRNA bearing cells were detected in peripheral blood before biopsy became undetectable after the biopsy. In Figure 2, representative RT-PCR findings from 4 PCa patients and 2 BPH patients are shown. PSA PCR product bands both before and after the biopsy were observed in Pt. No. 1, and 26. Pathological diagnosis of these were PCa, both of which had distant metastases and elevated sPSA of 380 ng ml$^{-1}$ and 376.8 ng ml$^{-1}$, respectively (Figure 2).

### Comparison of clinicopathological parameters and PSA RT-PCR assay findings

The 3 cases with detectable PSA-mRNA before biopsy were all PCa with extraprostatic disease (T3 or T4 and/or N1 and/or M1) and had higher sPSA levels and PSA density (PSAD) than the other 105 cases with negative RT-PCR assay ($P < 0.001$) (Figure 3A, B). The patients with positive PSA-mRNA after biopsy either likely had higher sPSA levels than those with negative mRNA (77.4 ng ml$^{-1}$ versus 11.3 ng ml$^{-1}$, $P < 0.05$), or PSAD levels (1.72 ng ml$^{-1}$ cm$^{-3}$ versus 0.35 ng ml$^{-1}$ cm$^{-3}$, $P < 0.05$) (Figure 3C, D). A significant difference between the proportion of positive PSA-mRNA among PCa cases (52.2%) and BPH cases (25.8%) was observed after biopsy, and this significance could also be observed between these 2 groups with gray zone sPSA (4.0–10.0 ng ml$^{-1}$) (Figure 4).

The clinical stages and PSA RT-PCR findings of 46 PCa patients are presented in Table 2. A significant difference between the proportion of positive PSA-mRNA among the PCa cases with extraprostatic disease (3 of 11 cases – 27.3%) and organ confined disease (T1 or T2, N0, M0) (0%) was observed before biopsy ($P < 0.01$) (Table 2), but other clinical tumour stages were not correlated with RT-PCR findings. Among 46 PCa patients, no correlation was observed between positivity of RT-PCR and pathological differentiation, grade or Gleason score, but positive PSA-mRNA cases had more cancer involved biopsy cores (mean 4.2) than negative PSA-mRNA cases (mean 2.2) after biopsy, and the difference was significant ($P < 0.001$) (Figure 5).

All 46 PCa patients were treated with combined androgen blockade following diagnostic procedures involving prostate biopsy, and 6 and 2 cases of these underwent radical prostatectomy and radiation therapy, respectively. 1 case with positive PSA-mRNA after biopsy and 1 with negative mRNA, died of disease-specific causes. Disease progressions showing new metastatic sites were identified in 5 cases with positive PSA-mRNA and one with negative mRNA with imaging such as CT or isotope bone scans. The disease progression-free survival rate was lower in the N0, M0 cases with positive PSA-mRNA after biopsy than in the N0, M0 cases with negative mRNA, however, the difference was not significant (Figure 6).

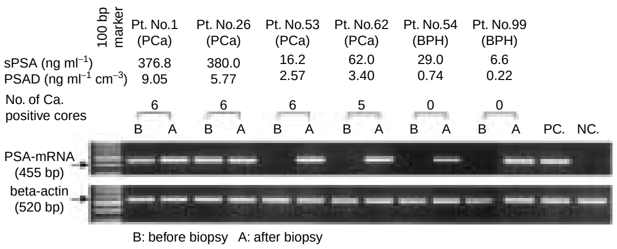

**Figure 2** Representative findings of nested RT-PCR. Pt. No. 1 and Pt. No. 26 are PCa cases with distant metastasis. Pt. No. 53 and Pt. No. 62 are PCa cases without distant metastasis. Pt. No. 54 and Pt. No. 99 are BPH cases. PC and NC are positive and negative controls

**Table 2** Summary of clinical stages of PCa cases and PSA RT-PCR findings

| | **PSA-mRNA (+) before biopsy** | **PSA-mRNA (+) after biopsy** |
|---|---|---|
| Organ confined disease (*n* = 35) | 0/35* | 16/35 |
| T1 (*n* = 22) | 0/22* | 4/22 |
| T2 (*n* = 13) | 0/13* | 12/13 |
| Extraprostatic disease (*n* = 11) | 3/11* | 8/11 |
| T3 (*n* = 2) | 1/2 | 2/2 |
| T4 (*n* = 1) | 0/1 | 1/1 |
| N1 and/or M1 (*n* = 8) | 2/8 | 5/8 |
| Total (*n* = 46) | 3/46 | 24/46 |

*$P < 0.01$.

### DISCUSSION

Recent studies showed tumour cells circulating in peripheral blood were observed in several cancers including RCC or breast cancer, and the consequence in disease staging or prognosis has been demonstrated (de la Taille et al 2000; Sabbatini et al 2000). In the present study, PSA-mRNA-bearing cells circulating in peripheral blood before prostate biopsy were identified in 3 of 11 cases (27.3%) of PCa with extraprostatic disease. These findings suggest that detection of circulating prostate cells would enable molecular staging in patients with PCa. However, 8 cases with extraprostatic disease including 1 patient with clinical stage T4, N0, M0 and 1121 ng ml$^{-1}$ of sPSA showed negative RT-PCR before biopsy. New strategies for accurate staging of the disease have been tried including flowcytometric detection of PSA-positive cells in peripheral blood (Eskola et al, 1994). RT-PCR is the most sensitive method and applicable to the molecular staging (Corey et al, 1998; Okegawa et al, 2000). Moreno et al, first applied PCR technology to the identification of circulating PSA-mRNA signals in patients with metastatic PCa and demonstrated a sensitivity of 33% (Moreno et al, 1992). Although detection rates of PSA-mRNA positive cells in peripheral blood of prostate cancer patients varied between 0 and 88%, with more accurate molecular biological techniques staging before surgery may be achieved. However, no standard RT-PCR procedure has been established for detecting PSA-mRNA-bearing cells in peripheral blood. The difference depends upon sample collection and fractionation, storage, reverse transcription and amplification.

The present RT-PCR detected the presence of PSA-mRNA in peripheral blood in 40 of 108 (37.0%) patients after sextant prostate biopsy using TRUS. Among them, 37 patients (34.3%) were negative for PSA RT-PCR before biopsy. Moreno et al, reported among 43 TRUS cases, 42 cases were negative before biopsy and 4 cases (9.5%) converted to a positive RT-PCR PSA findings (Moreno et al, 1997). Although the present methods for sample collection and RNA extraction differed from those of Moreno et al, the possibility of tumour spreading by prostate biopsy appears to be greater than that of previous studies.

© 2001 Cancer Research Campaign

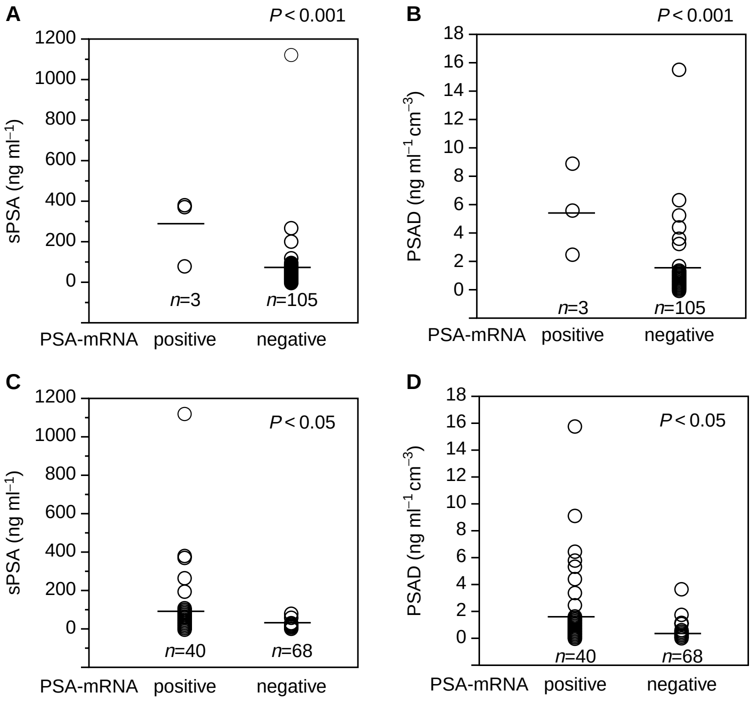

**Figure 3** (**A**) sPSA level in the PSA RT-PCR-positive and -negative groups before biopsy. (**B**) PSAD in the RT-PCR-positive and -negative groups before biopsy. (**C**) sPSA level in the RT-PCR-positive and -negative groups after biopsy. (**D**) PSAD in the RT-PCR-positive and -negative groups after biopsy

The present study showed that the detection rate of PSA-mRNA after biopsy was higher in those with elevated sPSA ($P < 0.05$) or PSAD ($P < 0.05$) indicating a higher likelihood of the presence of PCa. Indeed, the detection rate was higher in the PCa group ($P < 0.01$) and, the patients with more positive cores had more frequency of PSA-mRNA-bearing cells in peripheral blood ($P < 0.001$). This finding suggests that PSA-mRNA after biopsy might have a diagnostic value, and especially with higher volume cancer cases, prostate biopsy might scatter the prostate constructing cells or prostate cancer cells into the blood stream, which might contribute to micrometastasis. However, PSA-mRNA were detected in also non-PCa cases indicating the presence of PSA-mRNA does not necessarily mean the presence of prostate cancer cells in peripheral blood.

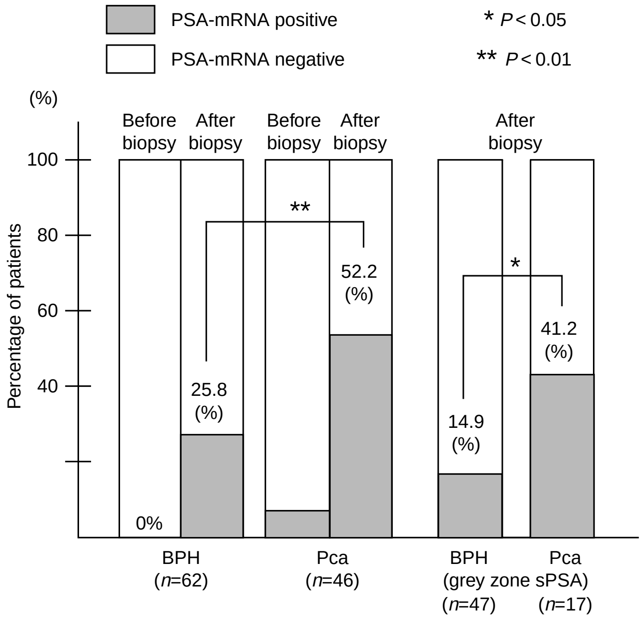

**Figure 4** Proportion of positive PSA RT-PCR cases before and after biopsy, and proportion of positive PSA RT-PCR cases after prostate biopsy in patients with grey zone sPSA (4.0–10.0 ng ml−1)

Implantation of tumour cells along the needle tract or dissemination in patients with cancer is a rare but potential complication

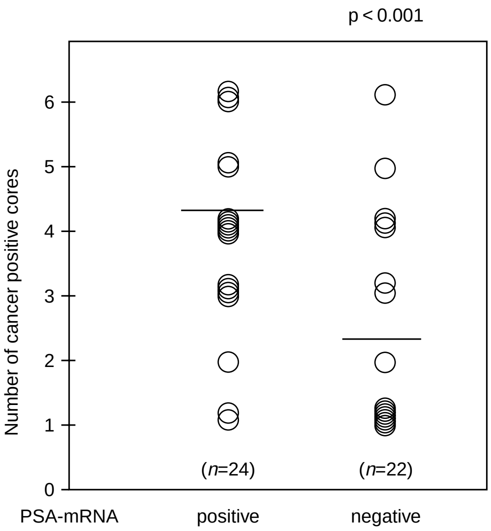

**Figure 5** Correlation of number of cancer positive cores and PSA RT-PCR findings after biopsy in PCa patients

© 2001 Cancer Research Campaign

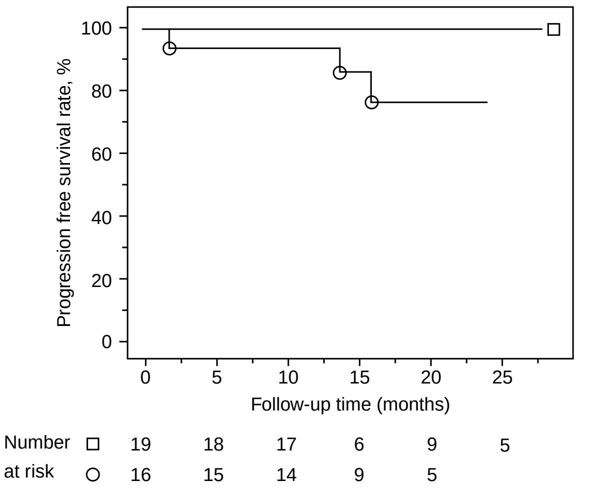

**Figure 6** Disease progression-free survival of N0, M0 cases categorized according to PSA-mRNA after prostate biopsy. □: group with negative PSA-mRNA after biopsy; ○: group with positive PSA-mRNA after biopsy following percutaneous needle biopsy, and was reported as chest wall implantation of lung cancer and breast cancer after fine needle aspiration biopsy (Voravud et al, 1992; Paik et al, 1994), and also as tumour extension along a biopsy needle tract from RCC (Smith et al, 1984; Kiser et al, 1986). However, it is still controversial whether surgical manipulation or biopsy of the prostate causes dissemination and/or micrometastasis of prostate cancer cells. RT-PCR is an extremely sensitive method to detect specific mRNA-bearing cells in the blood stream. It can detect a single specific mRNA-bearing cell among $10–10^8$ cells (Raj et al, 1998). Some studies reported that at least $10^4$ circulating tumour cells are required for metastasis (Liotta et al, 1976). Moreover, only 0.01% of circulating cancer cells create a single metastasis (Fidler, 1990; Fadlon et al, 1996). Thus, a correlation between prognosis and detection of cancer cells in blood after surgery or biopsy are needed to clarify their clinical significance. Although the clinico-pathological background and tumour biological behaviour in patients in the current study varied, the PSA-mRNA positive group after biopsy demonstrated a tendency towards decreased disease progression-free survival, indicating PSA-mRNA in blood after biopsy might have a prognostic value of disease progression-free survival. A long observation period is required to draw a definite conclusion and another modality to improve the information such as quantitative RT-PCR appears to be necessary for estimating the correlation between the number of circulating prostate cells and the presence of metastases or further disease progression after the biopsy.

It is concluded that nested RT-PCR could detect tumour cells in peripheral blood with higher sensitivity prior to metastases, enabling molecular staging of the tumour. We have also demonstrated that TRUS prostate biopsy might disperse prostate cells into the blood stream especially in cases with high sPSA. The prognosis for patients with clinically localized PCa and a positive PSA RT-PCR is currently not known. Although a prospective study comparing with the clinical course is required, to avoid dissemination of the tumour cells a minimal number of core biopsies for clinically and firmly diagnosed PCa should be adequate. To estimate the significance of detection of PSA-mRNA-bearing cells in the peripheral blood for metastasis and survival, further studies are warranted.

### REFERENCES

Berteau P, Gala JL, Eschwege P, Dumas F and Loric S (1999) The value of a reverse transcriptase polymerase chain reaction assay in preoperative staging and followup of patients with prostate cancer. *J Urol* **161**: 924–926

Catalona WJ, Richie JP, Ahmann FR, Hudson MA, Scardino PT, Flanigan RC, deKernion JB, Ratliff TL, Kavoussi LR and Dalkin BL (1994) Comparison of digital rectal examination and serum prostate specific antigen in the early detection of prostate cancer: results of a multicenter clinical trial of 6,630 men. *J Urol* **151**: 1283–1290

Corey E and Corey MJ (1998) Detection of disseminated prostate cells by reverse transcription-polymerase chain reaction (RT-PCR): technical and clinical aspects. *Int J Cancer* **77**: 655–673

Corey E, Arfman EW, Oswin MM, Melchior SW, Tindall DJ, Young CY, Ellis WJ and Vessella RL (1998) Detection of circulating prostate cells by reverse transcriptase-polymerase chain reaction of human glandular kallikrein (hK2) and prostate-specific antigen (PSA) messages. *Int J Cancer* **77**: 655–673

Crawford ED and DeAntoni EP (1993) PSA as a screening test for prostate cancer. *Urol Clin North Am* **20**: 637–646

Crawford ED, Bennett CL, Stone NN, Knight SJ, DeAntoni E, Sharp L, Garnick MB and Porterfield HA (1997) Comparison of perspectives on prostate cancer: analyses of survey data. *Urology* **50**: 366–372

de la Taille A, Olsson CA, Buttyan R, Benson MC, Bagiella E, Cao Y, Burchardt M, Chopin DK and Katz AE (1999) Blood-based reverse transcriptase polymerase chain reaction assays for prostatic specific antigen: long term follow-up confirms the potential utility of this assay in identifying patients more likely to have biochemical recurrence (rising PSA) following radical prostatectomy. *Int J Cancer* **84**: 360–364

de la Taille A, Katz A, Cao Y, McKiernan J, Buttyan R, Burchardt M, Burchardt T, Hayek O, Olsson CA, Chopin DK and Sawczuk IS (2000) Blood-based RT-PCR assays of MN/CA9 or PSMA: clinical application in renal cancer patients. *Urology* **56**: 393–398

Eskola JU, Hamalainen M, Nanto V, Rajamaki A, Dahlen P, Iitia A and Siitari H (1994) Detection of Philadelphia chromosome using PCR and europium-labeled DNA probes. *Clin Biochem* **27**: 373–379

Fadlon EJ and Hamdy FC (1998) The use of flow cytometry and RT-PCR in the detection of circulating PSA-positive cells in prostate cancer. *Method Mol Biol* **92**: 215–225

Fadlon EJ, Rees RC, McIntyre C, Sharrard RM, Lawry J and Hamdy FC (1996) Detection of circulating prostate-specific antigen-positive cells in patients with prostate cancer by flow cytometry and reverse transcription polymerase chain reaction. *Br J Cancer* **74**: 400–405

Ferrari AC, Stone NN, Eyler JN, Gao M, Mandeli J, Unger P, Gallagher RE and Stock R (1997) Prospective analysis of prostate-specific markers in pelvic lymph nodes of patients with high-risk prostate cancer. *J Natl Cancer Inst* **89**: 1498–1504

Fidler IJ (1990) Critical factors in the biology of human cancer metastasis: twenty-eighth G.H.A. Clowes memorial award lecture. *Cancer Res* **50**: 6130–6138

Gomella LG, Raj GV and Moreno JG (1997) Reverse transcriptase polymerase chain reaction for prostate specific antigen in the management of prostate cancer. *J Urol* **158**: 326–337

Greenlee RT, Murray T and Thun M (2001) Cancer statistics, 2001. *Cancer J Clin* **51**: 15–36

Kiser GC, Totonchy M and Barry JM (1986) Needle tract seeding after percutaneous renal adenocarcinoma aspiration. *J Urol* **136**: 1292–1293

Levine ES, Cisek VJ, Mulvihill MN and Cohen EL (1986) Role of transurethral resection in dissemination of cancer of prostate. *Urology* **28**: 179–183

Liotta LA, Saidel MG and Kleinerman J (1976) The significance of hematogenous tumor cell clumps in the metastatic process. *Cancer Res* **36**: 889–894

Mejean A, Vona G, Nalpas B, Damotte D, Brousse N, Chretien Y, Dufour B, Lacour B, Brechot C and Paterlini-Brechot P (2000) Detection of circulating prostate derived cells in patients with prostate adenocarcinoma is an independent risk factor for tumor recurrence. *J Urol* **163**: 2022–2029

Moreno JG, Croce CM, Fischer R, Monne M, Vihko P, Mulholland SG and Gomella LG (1992) Detection of hematogenous micrometastasis in patients with prostate cancer. *Cancer Res* **52**: 6110–6112

Moreno JG, O'Hara SM, Long JP, Veltri RW, Ning X, Alexander AA and Gomella LG (1997) Transrectal ultrasound-guided biopsy causes hematogenous dissemination of prostate cells as determined by RT-PCR. *Urology* **49**: 515–520

Okegawa T, Noda H, Kato M, Miyata A, Nutahara K and Higashihara E (2000) Value of reverse transcription polymerase chain reaction assay in pathological stage T3N0 prostate cancer. *Prostate* **44**: 210–218

Paik HC, Lee DY, Lee HK, Kim SJ and Lee KB (1994) Chest wall implantation of carcinoma after fine needle aspiration biopsy. *Yonsei Medical Journal* **35**: 349–354

*© 2001 Cancer Research Campaign* 

Raj GV, Moreno JG and Gomella LG (1998) Utilization of polymerase chain reaction technology in the detection of solid tumors. *Cancer* **82**: 1419–1442

Sabbatini R, Federico M, Morselli M, Depenni R, Cagossi K, Luppi M, Torelli G and Silingardi V (2000) Detection of circulating tumor cells by reverse transcriptase polymerase chain reaction of maspin in patients with breast cancer undergoing conventional-dose chemotherapy. *J Clin Oncol* **18**: 1914–1920

Saito T, Kimura M, Kawasaki T, Sato S and Tomita Y (1996) Correlation between integrin alpha 5 expression and the malignant phenotype of transitional cell carcinoma. *Br J Cancer* **73**: 327–331

Smith EH (1984) The hazards of fine-needle aspiration biopsy. *Ultrasound Med Biol* **10**: 629–634

Su SL, Boynton AL, Holmes EH, Elgamal AA and Murphy GP (2000) Detection of extraprostatic prostate cells utilizing reverse transcription-polymerase chain reaction. *Semin Surg Oncol* **18**: 17–28

Voravud N, Shin DM, Dekmezian RH, Dimery I, Lee JS and Hong WK (1992) Implantation metastasis of carcinoma after percutaneous fine-needle aspiration biopsy. *Chest* **102**: 313–315

Webber MM, Bello D, Kleinman HK, Wartinger DD, Williams DE and Rhim JS (1996) Prostate specific antigen and androgen receptor induction and characterization of an immortalized adult human prostatic epithelial cell line. *Carcinogenesis* **17**: 1641–1646

Yamamoto S (1999) Cancer statistics digest. Comparison of crude and age-standarized death rates in Japan. *Jpn J Clin Oncol* **29**: 649

Yuan JJ, Coplen DE, Petros JA, Figenshau RS, Ratliff TL, Smith DS and Catalona WJ (1992) Effects of rectal examination, prostatic massage, ultrasonography and needle biopsy on serum prostate specific antigen levels. *J Urol* **147**: 810–814

Zisman A, Soffer Y, Siegel YI, Paz A and Lindner A (1997) Post ejaculation serum prostate-specific antigen level. *Eur Urol* **32**: 54–57

 © 2001 Cancer Research Campaign
